# Prevalence of ABO and Rhesus (D) Blood Group and Allelic Frequency at Blood Bank of Nigist Eleni Mohammed Hospital, Ethiopia

**DOI:** 10.1155/2024/5353528

**Published:** 2024-04-08

**Authors:** Fekadu Lendabo, Vijayalakshmi Srinivasan, Riyaz Ahmad Rather

**Affiliations:** ^1^Department of Biotechnology, College of Natural and Computational Science, Wachemo University, Hossana, Ethiopia; ^2^M.Ct.M Chidambaram Chettyar International School, Chennai, India

## Abstract

**Background:**

The purpose of this cross-sectional study was to determine the pattern of the ABO and rhesus D (RhD) blood group distribution among voluntary blood donors attending five blood donation centers at Nigist Eleni Mohammed General Hospital in Hossana, Ethiopia.

**Methods:**

A total of 1,120 participants who fulfilled the “who can give blood” criteria of the World Health Organization were selected randomly. Blood samples were collected, transported to the laboratory, and analyzed for ABO and RhD typing. The data was analyzed using descriptive statistics and chi-square correlation analysis.

**Results:**

The study found that the O blood group was the most prevalent (39.0%), followed by A (32.2%), B (22.5%), and AB (6.4%). When considering both the ABO and Rh blood groups together, 92.9% of blood donors were RhD positive, while only 7.1% were RhD negative. The distribution pattern of the ABO blood groups in Gurage Zone, Hadiya Zone, Kembata Zone, and Silte Zone showed that the O blood group was the most prevalent, followed by A, B, and AB, in that order. Conversely, the ABO blood group distribution pattern in Halaba Zone was A > O > B > AB. Civil servants from different occupational statuses were the most dominant voluntary blood donors, accounting for 53.2%, followed by students from different high schools and universities (41.9%), self-employed individuals (4.1%), and others (0.7%). The ABO blood group system had observed allele frequencies significantly different from the expected frequencies (*p* = 0.007), while the RhD system did not (*p* = 0.037). Allele frequencies for A, B, and O in the ABO system were 0.3531, 0.2576, and 0.3893, respectively. Observed frequencies for RhD-positive and RhD-negative alleles were 0.9647 and 0.0531, respectively.

**Conclusion:**

This study highlights the regional ABO and RhD blood group variations in Ethiopia, noting disparities from expected ABO allele frequencies, and identifies the O blood group predominance among donors with a high RhD-positive prevalence.

## 1. Introduction

The discovery of the blood group system marked a significant turning point in transfusion medicine by making blood transfusions safe and effective. Blood group phenotyping is important in transfusion medicine and has important clinical implications [1]. With numerous blood groups identified in humans to date, the ABO and Rh blood group system holds particular significance in transfusion medicine. At the molecular level, the ABO system exhibits three alleles in the ABO genes, I^A^, I^B^, and I^O^, leading to six potential genotypes within the population—I^A^I^A^, I^A^I^O^, I^B^I^B^, I^B^I^O^, I^A^I^B^, and I^O^I^O^. This results in four distinct ABO phenotypes: A blood group (I^A^I^A^ and I^A^I^O^), B blood group (I^B^I^B^ and I^B^I^O^), AB blood group (I^A^I^B^), and O blood group (I^O^I^O^) [2]. Rhesus D- (RhD-) positive individuals have genotypes I^D^I^D^ or I^D^I^d^, whereas RhD-negative individuals have the genotype I^d^I^d^ [3]. The ABO blood group system is controlled by a single gene at the ABO locus on chromosome 9 at 9q34.1-q34.2. This locus comprises seven exons, spanning over 18 kb of genomic DNA. The largest among them is exon 7, housing the majority of the coding sequence [4]. Exons encode a glycosyltransferase enzyme, which adds a sugar residue to the H antigen, which is found in the membranes of red cells as well as most epithelial and endothelial cells. The A allele codes for an enzyme that adds N-acetyl galactosamine to the H antigen, whereas the B allele, which differs by four amino acid modifications, codes for an enzyme that attaches D-galactose [5]. The O allele, which is more common in humans, carries a human-specific inactivating mutation, resulting in a nonfunctional enzyme and the retention of the H antigen on the cell surface without further modification [6]. The ABO blood group system is hence determined by the presence or absence of A and B antigens on the surface of red blood cells. In contrast, the RhD blood group system is based on the D antigen, which is present in approximately 85% of the general population. The RhD gene, responsible for encoding RhD proteins (D antigen), is situated on chromosome 1p34-p36.31. Spanning 69 kb of DNA, this gene is comprised of 10 exons and originated from a gene duplication event [7]. The ABO and RhD blood groups are the most significant in transfusion practice, and understanding their frequency and distribution among blood donors is critical for the safe and effective delivery of transfusion services. Mismatched blood can cause major adverse reactions, including hemolytic reactions that can destroy RBCs and even result in death [8].

In recent years, more research has focused on the prevalence and distribution of the ABO and RhD blood group phenotypes among blood donors from various populations. These findings suggest that the blood group phenotypic frequency varies greatly across ethnic groups, geographical areas, and populations [9, 10]. Understanding the phenotypic characteristics of blood groups in Ethiopia, a country with many different ethnic groups and a diverse population, is critical for providing efficient and secure transfusion services. Recently, research studies have looked into the prevalence and distribution of different blood group phenotypes in Ethiopia, but the majority of these studies have a narrow focus on specific areas or groups [11, 12]. As a result, the current study was conducted to examine various blood phenotypes and allele frequencies among people who donate blood at various donation centers of Nigist Eleni Mohammed General Hospital (NEMGH), Hossana, Ethiopia.

## 2. Materials and Methods

The study was carried out at the blood bank of NEMGH from March 2021 to December 2022. NEMGH is a tertiary care hospital in Hossana, a town 245 km to the south of Addis Ababa. It coordinates blood banks that are located in Hadiya Zone, Gurage Zone, Halaba Zone, Kembata Zone, and Silte Zone. Individuals who voluntarily donated blood at these centers and were willing to participate were randomly selected for this study. Informed consent was acquired from the study participants, and the research was approved by the Ethics Board of Wachemo University under memo with ID number EBC/WC/352/2021. This study employed only those donors who met the World Health Organization's “who can give blood” criteria [13]. The single population proportion formula was used with a proportion of 50% to determine the required sample size. Additionally, a 95% confidence interval and a 5% margin of error for an inconvenient sample were taken into account during the determination of the sample size. The required sample size was determined to be 1,067. The total sample size was calculated as 1,120 with a 5% margin of error.

During a blood donation, from each individual, a total of 3 ml of blood was collected from each unit of donated blood. The blood was collected in an EDTA vial, kept in a cooling box at 4°C, and transported to the hematology laboratory of the NEMGH. In the laboratory, the blood samples were brought to room temperature. ABO and RhD typing was performed by slide agglutination test using commercially available antisera, anti-A, anti-B, and anti-D (Bio Lab Diagnostics, Mumbai, India). Briefly, a drop of blood was placed at three different places on the clean slide. To each blood spot, a drop of the respective antisera was added, mixed with separate sterilized applicator sticks, and observed for agglutination with the naked eye or microscope. Based on the Hardy-Weinberg equilibrium, the allele frequencies of the ABO and RhD blood group genes were calculated using the following equations:

(1) $$\begin{array}{c} p=1-\sqrt{B}+O,\\ q=1-\sqrt{A}+O,\\ r=\sqrt{O},\\ E=1-e,\\ e\sqrt{\text{dd}}. \end{array}$$

Here, *p*, *q*, *r*, *E*, and *e* represent the gene allele frequencies for A, B, O, RhD positive, and RhD negative, respectively.

### 2.1. Statistical Analysis

For data analysis, all donor ABO and RhD blood groups were tabulated in Microsoft Excel 2016 version. Demographic variables associated with the study participants were recorded in a data sheet. The data were analyzed using descriptive statistics, which included absolute frequencies and percentages. Chi-square correlation analysis was used to determine the differences in blood group phenotypes. With a 95% confidence level, *p* ≤ 0.05 was considered statistically significant.

## 3. Results

The majority of voluntary blood donors were males (68.2%), and the highest number (49.8%) of donors was within the age range of 18-24. Most blood donors were from the Gurage Zone (25.9%), followed by the Hadiya Zone (23.2%). Kembata Zone had the least voluntary blood donors (18.7%), with the Silte Zone following the lowest (10.5%). Civil servants from different occupational statuses were the most dominant voluntary blood donors, accounting for 53.2%, followed by students from different high schools and universities (41.9%), self-employed individuals (4.1%), and others (0.7%) (Table 1).

**Figure 1:**
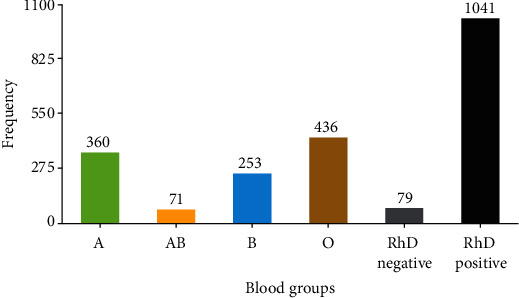
Prevalence of the ABO and RhD blood group phenotype of study participants. The percentages presented represent the proportion of each variable among the total study participants (*n* = 1,120).

The study found that the O blood group was the most prevalent (39.0%), followed by A (32.1%), B (22.5%), and AB (6.4%). When considering both the ABO and Rh blood groups together, 92.9% of blood donors were RhD positive, while only 7.1% were RhD negative (Figure 1). In a comparison of all the RhD-positive and RhD-negative blood groups, the most prevalent blood group was O positive (36.3%), followed by A positive (29.6%), B positive (21.0%), and AB positive (6.0%). Both O negative and A negative were equally prevalent and more prevalent than other RhD-negative blood groups, which accounted for 2.6%, followed by B negative (1.5%) and AB negative (0.4%) (Table 2).

In terms of ABO blood group distribution patterns at blood donation sites, the O blood group was the most prevalent (9.7%) and had an equal distribution in the majority of the blood donation sites, including Gurage Zone, Hadiya Zone, and Kembata Zone. However, the AB blood group had the smallest distribution pattern in the Silte Zone (0.4%) compared to the others. The distribution pattern of the ABO blood groups in Gurage Zone, Hadiya Zone, Kembata Zone, and Silte Zone showed that the O blood group was the most prevalent, followed by A, B, and AB, in that order, when compared across all blood donation sites. In contrast, the ABO blood group distribution pattern in Halaba Zone was A > O > B > AB (Table 3). RhD-negative blood types were more prevalent and evenly distributed in Halaba Zone and Kembata Zone (2.23%), with an equal distribution in Gurage Zone and Hadiya Zone (1.1%) and the least frequency in Silte Zone (0.4%). On the other hand, RhD-positive blood types were more prevalent in Gurage Zone (24.8%), Hadiya Zone (22.0%), Halaba Zone (19.5%), Kembata Zone (16.5%), and Silte Zone (10.1%).

**Figure 2:**
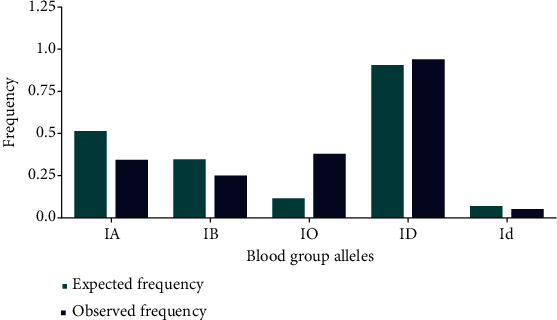
The expected and the observed allele frequencies in the blood group system of study participants. IA, IB, IO, ID, and Id refer to the I^A^, I^B^, I^O^, I^D^, and I^d^ blood group alleles of ABO and RhD system, respectively.

Allele frequencies for the ABO blood group system were 0.527 for the A allele, 0.355 for the B allele, and 0.118 for the O allele. The observed allele frequencies were 0.3531 for the A allele, 0.2576 for the B allele, and 0.3893 for the O allele (*χ*^2^ = 11.5, df = 2, *p* = 0.007) (Figure 2).

The expected allele frequency for the RhD system was 0.929 for the RhD-positive allele and 0.071 for the RhD-negative allele, while the observed allele frequencies were 0.9647 for the RhD-positive allele and 0.0531 for the RhD-negative allele (*χ*^2^ = 0.068, df = 1, *p* = 0.037) (Figure 2). Our analysis shows that the observed allele frequencies in the ABO blood group system are significantly different (*p* = 0.007) from the expected frequencies, while there is no significant difference (*p* = 0.083) in the observed and expected allele frequencies in the RhD system.

## 4. Discussion

According to the study's findings, men made up 68.2% of voluntary blood donors, which is consistent with research from other Ethiopian studies [14–16]. In other trials across Africa, including Ethiopia, 67.8% of donors were men, compared to 32.2% of women in one study and 55.8% of men and 44.2% of women in another [17–19]. These findings could be explained by sociocultural or religious beliefs, nursing and pregnancy among female donors, and their aversion to giving blood in public. It is critical to encourage more women to donate blood in order to ensure a consistent and ample blood supply. The study also found that the majority of donors (49.8%) were between the ages of 18 and 24. Age and interest in blood donation were found to be inversely correlated in our study. This finding is consistent with previous research and findings from other regions of Ethiopia [14, 20]. Younger people may be more interested in and capable of donating blood because they are more aware, have better physical health, and are more mobile. Elderly people, on the other hand, may have health issues such as ischemic heart disease, diabetes mellitus, cancer, and hypertension, which may limit their ability to donate blood.

The study's findings demonstrate that the phenotypic frequency distribution of the ABO and RhD blood types, as well as the number of volunteers who donate blood at each donation station, fluctuates significantly across various environmental contexts and might not be equally distributed in various populations. The various distribution patterns of donors at various blood donation places may be explained by the township's way of life. People in the Gurage and Hadiya zones, for instance, can visit a nearby blood bank, which promotes the act of donating blood. On the other hand, there is not a nearby blood bank in the Halaba Zone and Silte Zone neighborhoods. There were fewer study participants from these regions who were seen fasting during the time when samples were being collected. These regions have the lowest number of blood donors because the vast majority of residents adhere to the Islamic faith, which makes it challenging for them to donate blood willingly. A number of variables [21–25] ultimately influence the frequency of blood donations. The majority of participants in the current study who donated blood were civil servants. However, one study conducted by Lownik et al. suggests that students were more likely to give blood voluntarily [26]. Although the precise determinants of this observed contrast were not the primary focus of our study, some plausible explanations can be inferred. One possible reason for the high blood donation frequency among civil servants is their potential exposure to healthcare contexts due to the nature of their work, which may foster a better understanding of the benefits associated with blood donation, thereby driving their active participation. On the other side, students' lesser participation in blood donation could be attributed to their relaxed attitude towards the activity. This attitude may be rooted in a lack of information about the significance of blood donation or a belief that it is not a pressing priority in their lives. Besides that, the dynamics of blood donation engagement are likely influenced by factors such as cultural norms, educational background, and personal motivations. Additional research is required to precisely identify the factors influencing blood donation practices among donors. A more comprehensive analysis of these characteristics is needed to gain a deeper understanding of the variations in blood donation patterns across different demographic groups.

In terms of ABO phenotype, the current study participants' blood types were O > A > B > AB (39.0% vs. 32.2 vs. 22.5% vs. 6.4%), while RhD phenotype was RhD positive > RhD negative (92.9% vs. 7.1%). The prevalence of the ABO and RhD blood groups varies greatly around the globe and even among ethnic groups within the same geographic location. Several studies have shown that the O blood group is the most frequent, whereas the AB blood group is the least prevalent among various ethnic groups. In one study, for example, the predominance of the ABO and Rh blood groups in the Silte Zone was discovered to be O (41.0%), A (24.5%), B (21.3%), and AB (5.2%), with Rh factors of 92.06% RhD positive and 7.94% RhD negative [27]. Similarly, Enawgaw et al. found that in Ethiopia's Amhara regional state, the predominance of ABO and Rh blood groups was O (41.6%) > A (28.7%) > B (22.2%) > AB (7.7%), with RhD positive (92.5%) > RhD negative (7.5%) [28]. Our findings are consistent with those of research conducted in Ethiopia and elsewhere [29–34] (Table 4). According to our findings, the distribution of the ABO blood groups within the Halaba Zone follows a specific pattern: A > O > B > AB. This distribution deviates from some of the more commonly seen patterns in other groups and locales.

The prevalence of the A blood group in the Halaba Zone contrasts with the internationally more widespread O blood group. This variety may represent distinct genetic and evolutionary features unique to the population in this location. More genetic research could reveal whether this distribution is the product of historical genetic isolation or of a distinct selecting pressure that favored the A blood group. The O blood group has a higher frequency than the B and AB blood groups, which corresponds to broader global trends. Because of its compatibility with various blood types, the O blood group is frequently referred to as a universal donor, making it extremely helpful in medical contexts. The presence of a substantial O blood group proportion could have implications for blood donation practices and transfusion services within the Halaba Zone. The lower occurrence of the B and AB blood groups might be a reflection of limited gene flow from neighboring populations that possess higher proportions of these blood groups. This could point towards the region's genetic distinctiveness and potential historical factors that have shaped the genetic composition of its population. Additionally, the study found that 92.9% of blood donors were RhD positive, while only 7.1% were RhD negative, which is in agreement with studies conducted elsewhere. For instance, in a study conducted in Debre Tabor, Ethiopia, the RhD-positive blood group was the most common (92.7%) blood group present in blood donors [35]. A higher prevalence of RhD positive has been reported in numerous studies conducted in Ethiopia and around the world in comparison to the RhD-negative phenotype [25, 26, 28, 30–32, 36–39]. In contrast to the prevalent trend, a study carried out in Gambela, Ethiopia, revealed a higher proportion (19.37%) of the RhD-negative blood groups compared to the prevalence observed in other studies conducted in different regions [38].

The results of the present study showed a significant difference in the observed allele frequencies for the ABO blood group system when compared to the expected frequencies. The observed allele frequencies for the ABO blood group system in the present study were 0.527 for the A allele, 0.355 for the B allele, and 0.118 for the O allele. Our findings align with the results of previous studies conducted in different parts of Ethiopia [39, 40]. The higher frequency of the O allele is consistent with the high prevalence of the O blood group, which is considered the most prevalent blood group worldwide. The difference in the observed allele frequencies in the ABO blood group system may be attributed to genetic variation, population structure, and natural selection. In contrast, there was no significant difference in the observed and expected allele frequencies for the RhD system. The observed allele frequencies for the RhD system in the present study were 0.9647 for the RHD-positive allele and 0.0531 for the RHD-negative allele, which are consistent with previous studies conducted in Ethiopia [29, 30].

The novelty of the results in this study lies in several key findings. Firstly, there is a notable increase in the prevalence of male donors (68.2%) and a concentration of donors within the 18-24 age bracket (49.8%), indicating changing trends in gender-based participation and effective strategies for engaging younger demographics. Secondly, the study reveals significant geographic disparities in blood donation rates across zones, with Gurage Zone and Hadiya Zone emerging as prominent contributors, suggesting potential variations in awareness campaigns, accessibility to donation centers, or cultural factors. Thirdly, the ABO blood group distribution pattern in the Halaba Zone, A > O > B > AB, deviates from the typical global pattern, warranting investigation into unique genetic or evolutionary influences shaping this distinct distribution. Fourthly, the even distribution of RhD-negative blood types in the Halaba Zone and Kembata Zone (2.23%) contrasts with the generally lower prevalence of RhD-negative types, raising questions about potential genetic drift, selection pressures, or historical factors impacting these genetic traits. Lastly, the study reports a significant difference in the observed allele frequencies in the ABO blood group system (*p* = 0.007) compared to expected frequencies and the observed allele frequencies for the RhD system (*p* = 0.037), suggesting potential genetic factors that need further exploration. These findings contribute substantially to our understanding of evolving blood donation practices, genetic diversity, and regional variations. They emphasize the need for further investigations to elucidate the specific factors driving these deviations and enable the tailoring of effective strategies for blood donation campaigns, healthcare services, and genetic studies within the region. While this study provides valuable insights into the ABO and RhD blood group distributions among voluntary blood donors, it is important to acknowledge its limitations. This study, conducted in a single blood bank with voluntary donors, has potential limitations including sampling bias and the absence of a broader cultural and socioeconomic context.

## 5. Conclusion

This study adds to our understanding of the ABO and RhD blood group distribution patterns among volunteer blood donors in Southern Ethiopia. According to the study's findings, the majority of donors were males aged 18 to 24, with the O blood group being the most prevalent, followed by A and B. While there was no significant difference between the actual and expected allele frequencies in the RhD system, the observed allele frequencies in the ABO blood type system were significantly different from the expected rates. When compared to previous research in the same region, the study's contrasting findings reveal shifts in male donor prevalence and age distribution, geographic variations in donation rates, and distinct ABO/RhD blood group patterns, providing novel insights into blood donation practices and genetic traits. These findings have far-reaching implications for public health policies and blood transfusion programs in the region. The findings of this study will help us better understand the phenotypic frequency and distribution of the ABO and RhD blood groups in Ethiopia. Furthermore, this research will provide light on the phenotypic characteristics of the local blood groups, as well as aid in the planning and administration of transfusion services at NEMGH and throughout Ethiopia. The necessity for conducting a future meta-analysis study that amalgamates data from all prior research, including the present study, is evident. Such an analysis will serve to interlink findings and offer a more comprehensive understanding of the overall prevalence of blood groups in the country. This undertaking holds the potential to provide invaluable insights into this crucial aspect of healthcare.

## Figures and Tables

**Table 1:** Sociodemographic characteristics of blood donors (*n* = 1,120).

Variables	Variable	Frequency (*n*)	Percentage
Sex	Female	356	31.8%
	Male	764	68.2%

Age	18-24	558	49.8%
	25-31	361	32.2%
	32-38	147	13.1%
	39-45	50	4.5%
	46-65	4	0.4%

Blood donation sites	Gurage Zone	290	25.9%
	Hadiya Zone	260	23.2%
	Halaba Zone	243	21.7%
	Kembata Zone	209	18.7%
	Silte Zone	118	10.5%

Occupation	Civil servants	596	53.2%
	Students	469	41.9%
	Self-employed	46	4.1%
	Others^∗^	9	0.8%

^∗^Others include those who did not reveal their occupation.

**Table 2:** Combined distribution pattern of the RhD and ABO blood group phenotype among study population (*n* = 1,120).

RhD types	ABO blood groups				Total (%)
	A (%)	AB (%)	B (%)	O (%)	
RhD negative	29 (2.6%)	4 (0.4%)	17 (1.5%)	29 (2.6%)	79 (7.1%)
RhD positive	331 (29.6%)	67 (6.0%)	236 (21.0%)	407 (36.3%)	1,041 (92.9%)
Total	360 (32.1%)	71 (6.4%)	253 (22.5%)	436 (36.0%)	1,120 (100%)

**Table 3:** Distribution pattern of the ABO blood group phenotype at different sites (*n* = 1,120).

ABO phenotype	Blood donation sites					Total
	Gurage Zone	Hadiya Zone	Halaba Zone	Kembata Zone	Silte Zone	
A	97 (8.6%)	71 (6.4%)	106 (9.4%)	53 (4.7%)	33 (3.0%)	360 (32.1%)
AB	17 (1.5%)	21 (1.9%)	17 (1.5%)	12 (1.1%)	4 (0.4%)	71 (6.4%)
B	68 (6.0%)	59 (5.3%)	55 (4.9%)	33 (3.0%)	38 (3.3%)	253 (22.5%)
O	109 (9.7%)	109 (9.7%)	67 (6.0%)	109 (9.7%)	42 (3.7%)	436 (39.0%)
*χ* ^2^ = 10.48, df = 4						

**Table 4:** ABO and Rh (D) blood group distribution among blood donors in Ethiopia, as predicted by certain studies^∗^.

ABO and Rh (D) blood group	Current study	Addis Ababa	Jimma	Bahir Dar	Gondar	Arba Minch	Jijiga	Debre Tabor
A	32.1%	28.4%	31.9%	29.8%	26.4%	32.7%	29.6%	29.8%
B	22.5%	21.3%	21.5%	23.2%	21.7%	20.9%	15.2%	23.2%
AB	6.4%	5.7%	3.5%	5.5%	4.9%	4.3%	5.1%	5.5%
O	39.0%	44.6%	43.1%	41.5%	47.0%	42.1%	50.2%	41.5%
Rh (D) positive	92.9%	94.8%	92.8%	91.5%	94.2%	92.8%	95.6%	91.5%
Rh (D) negative	7.1%	5.2%	7.2%	8.5%	5.8%	7.2%	4.4%	8.5%

^∗^Elements of the table have been incorporated from the source cited in reference [28].

## Data Availability

The data related to this study is available from the corresponding author.
